# Editorial Extracts and Gleanings

**Published:** 1874-02

**Authors:** T. Curtis Smith

**Affiliations:** Ohio


### EXTRACTS AND GLEANINGS

BY T. CURTIS SMITH, M.D., OF OHIO.

*Needle Swallowed by a Child—Removed Six Months Afterwards from the Thigh.*—By B. Schermerhorn, M.D., of Buskirk's Bridge, N. Y.—Emma S., a child of fourteen months, was brought to me June 18, 1873, by its parents, who gave the following history: About six months ago the child swallowed an ordinary sewing needle which lodged in the throat, and was pushed downward with a swab in the hands of its grandmother. From that time until about a month since it gave the child no trouble; since then it has been more or less worrisome and fretful, and during the last night before being brought to me it cried the whole time. Upon dressing the little one in the morning the mother's attention was drawn to a little elevation of the skin, like a pimple, upon the outer aspect of the left thigh, midway between the knee and hip-joint. Thinking of the needle they came with the child to my office, and after laying open the cuticle I removed a needle an inch and a quarter in length, which lay with its eye to the surface.

*Resection at Hip-joint; Fracture of Femur.*—Dr. F. H. Hamilton, at a recent meeting of the New York Pathological Society (*Med. Record*, August 1, 1878), presented the head and neck, and trochanter major of a femur resected at Bellevue Hospital before the class, April 12; 1873. The patient was a boy, æt. seven, who had morbus coxæ in its third stage. He was now progressing favourably toward recovery. The only point of special interest in the case was the fact that, while employing a slight amount of leverage to disarticulate the bone, the femur broke near its middle. He was surprised how little force was requisite to break the femur under these circumstances; and as he had not seen any allusion to this danger in the surgical treatises, he thought it important that he should call the attention of surgeons to the matter. The fracture was transverse, and under the support of pasteboard splints it united speedily without deformity.

Dr. Sayre said this accident had happened in two cases while he was operating.

*Croton-Chloral Hydrate.*—Dr. B. Barker, in the *British Medical Journal*, relates numerous cases in which this agent in cases suffering nerve pain, neuralgia. The dose given was one or two grains, with generally prompt relief. In various instances where the usual remedies for neuralgia had failed, the croton-chloral hydrate was prompt in affording relief. In thirteen cases where the agent had been resorted to for pain of this character, no bad sequential symptoms followed its use. It seems to produce natural sleep, and act also as an efficient anodyne. Occasionally it acted as a laxative.—*Reporter*.

*The Elastic Lignture.*—Dr. Dittel, of Vienna, has brought forward the elastic ligature for practical use in the removal of tumors, extremities, etc. He was led to use it by observing a case where death was caused by the elastic cord of a head net, having cut its way through the scalp, calvarium and into the brain. The patient died with obscure brain symptoms. At the necropsey, the cord as above described was found. After this he performed various operations which proved successful and bloodless, the ligature cutting its way through the soft tissues and bone, slowly but steadily and surely. By *The Clinic* for December 20, 1873, we learn that Sir Henry Thompson, of London, recently demonstrated the practicability of this procedure, by removing with the elastic ligature a mammary tumor. The operation consisted "in substituting an innocent-looking elastic thread for the formidable array of knives, tourniquets, artery forceps, and other paraphanalia with which the surgeon ordinarily approaches the patient." In Sir Henry Thompson's case, the tumor was a cystic sarcoma, of twenty years standing, which had recently enlarged so that the integument had given way, and "it was crowned by a large, sloughy, fungating ulcer." The elastic ligature was passed under the base of the tumor with a nævus needle and tied in halves separately. A stout twine was passed with the ligature so that in the event of the ligature breaking another could be drawn through. The patient complained of a little pain for twenty minutes after anesthesia ceased; otherwise no pain is mentioned. The ligature should be freshly prepared as it becomes to some extent brittle by age.

*Syphilitic Urethral Discharges.*—The last volume of the St. George's Hospital Reports contains three lectures on urethral discharges by Mr. Henry Lee. In the first he shows that syphilitic persons are liable to a urethral discharge which is not gonorrheal, and which may communicate syphilis to a virgin subject. Of these syphilitie urethral discharges there are two-thirds; the primary and the secondary. In the former the discharge is attended by circumscribed adhesive inflammation of the urethra, enlargement of the inguinal glands and constitutional infection; the second variety being sometimes seen in syphilitic persons after sexual intercourse. Mr. Lee shows that the discharge in both instances is capable of conveying syphilis, and contends that this is the rational explanation of many of the supposed cases of syphilis supervening on gonorrhea. He moreover asserts that it is highly probable that hereditary syphilis is more frequently transmitted by semen mixing with the products of syphilitic diseases of the urethra than by a morbid condition of the seminal fluid itself. In the second and third lecture he completely crushes the spermatorrheæists, by showing that the semen does not enter the vesicules seminales at all, and that in socalled spermetorrhea the discharge consists of the secretion of the vesiculæ. Further on he makes some suggestive remarks, on the treatment of stricture of the urethra, which is sometimes due to a syphilitic deposit in the urethral mucus membrane, and which is remediable only by constitutional treatment.—*Reporter.*

*Foreign Bodies in the Air Passages.*—Dr. W. W. Dawson, Prof. *Surgery Medical College of Ohio*, precedes the history of seven cases of this class by the following statement, which contain valuable information. "Mr. Durham, of London, has tabulated five hundred and fifty-four cases; of these two hundred and seventy-one were not operated upon. Two hundred and fifty-six of them recovered, one hundred and fifteen died, showing a mortality of forty-two, five per cent. In the remaining two hundred and eighty-eight, bronchotomy was performed, two hundred and thirteen recovered and seventy died, giving a motality for the operation of twenty-four, five per cent. Interference over noninterference, has here an advantage of seventeen per cent." "Dr. J. R. Weist, of Richmond, Indiana, has collected and analyzed one hundred and sixty-three cases of foreign bodies in the air passages; eighty-two were operated upon, eighty-one were allowed to take care of themselves." Dr. "Weist says, as determined by Prof. Gross' table, the chances for recovery are more than twice as great after bronechotomy as they are without this operation; while the cases here only show a difference of one and half per cent. in favor of the operation; and I feel sure from observations made during the collection of material for this paper, that were it possible to collect from medical men generally *all* the facts known to them in relation to this subject, the difference in favor of operation would be reduced still more.

Of the seven cases related by Prof. Dawson, two had bronchotomy performed, the foreign bodies removed. Both recovered. Of the other five cases, all of them excelled the foreign bodies spontaneously. Four of them recovered, one died.

The foreign bodies were in these seven cases, respectively, the "husk of a beechnut;" "half glass button," a "kidney bean," "piece of oyster shell," "pumpkin seed." "a shawl-pin two and a half inch long, which remained in the trachea five and a half months," the head of the pin being glass," "one-fourth of an inch in diameter, and last," "a puff-dart two and a quarter inches in length in the trachea for three months and eight days." This last case resulted fatally.—*The Clinic for January* 3, 1874.

*Ferruginous and Nerve Tonic.*—℞ Tr. Ferri chloridi ʒvi, ammonia hydrochlor ʒiii, aquæ mentha piper syr. autantei *a. a.* ℥iiiss. Mix S. Two to four drachms three times a day. It is pleasant and valuable in suitable cases. To the above may be added when needed, strichnine or quinine sulp., which makes it a tonic hard to surpass in value, as a general tonic, and especially where the system is under the influence of malaria.

---

